# Protocol for a randomized controlled trial of patient–portal–based screening for substance use among people with HIV

**DOI:** 10.3389/fpubh.2025.1583546

**Published:** 2025-08-20

**Authors:** Eric Roessler, Daniela Zimmer, Jon Grant, Harold Pollack, Basmattee Boodram, Jessica Schmitt, Eleanor Friedman, Jade Pagkas-Bather, Russell A. Brewer, Jessica Ridgway, Neda Laiteerapong

**Affiliations:** ^1^Department of Medicine, University of Chicago, Chicago, IL, United States; ^2^Department of Psychiatry and Behavioral Neuroscience, University of Chicago, Chicago, IL, United States; ^3^Crown Family School of Social Work, Policy, and Practice, Department of Public Health Sciences, and Urban Health Lab, University of Chicago, Chicago, IL, United States; ^4^Division of Community Health Sciences, School of Public Health, University of Illinois at Chicago, Chicago, IL, United States; ^5^Chicago Center for HIV Elimination, Department of Medicine, University of Chicago, Chicago, IL, United States

**Keywords:** substance use disorder, HIV, population health, patient portal screening, Comorbidity

## Abstract

**Background:**

Achieving Equity in Patient Outcome Reporting for Timely Assessments of Life with HIV and Substance Use (ePORTAL HIV-S) is a research project funded by the National Institute for Drug Abuse to implement and evaluate multi-level interventions to decrease barriers to substance use screening and treatment for PLWH. At its center is a multidomain intervention addressing digital, sociocultural, and health care system environments, at individual, interpersonal, and community levels. ePORTAL HIV-S has four overall goals; this manuscript describes the protocol specifically for the randomized control trial (RCT) portion of the study. To provide additional context, we briefly describe the overall ePORTAL HIV-S project.

**Methods:**

This project will utilize a culturally tailored approach to increase patient portal use among PLWH in our health system via a community health worker (CHW)-led initiative. This will lay the groundwork for the second aim, the focus of the current manuscript, RCT to measure the effectiveness of a population health, patient portal-based substance use screening program. Approximately 880 people will be enrolled and randomized 1:1 to intervention vs., control arms. Participants in the control arm will receive usual care (substance use screening during clinic visits), whereas the intervention arm will be invited to complete substance use screening via the patient portal as well as during clinic visits as per usual care. The primary outcome will be the percentage of people screened for substance use. ePORTAL will also implement a collaborative care model to both connect patients who screen positive for SUD to care and effectively treat PLWH. Finally, we will plan for dissemination of ePORTAL HIV-S to other sites that provide care for PLWH.

**Discussion:**

SUD disproportionately impacts PLWH which leads to negative health outcomes. This novel approach will incorporate the privacy and convenience of patient portal screening with screening during routine clinic visits.

**Clinical trial registration:**

clinicaltrials.gov, identifier NCT06682468.

## Introduction

Substance use disorders (SUDs) and HIV are synergistic epidemics in the United States. Having a SUD is a known risk factor for the acquisition of HIV (1), and many people living with HIV (PLWH) belong to historically marginalized groups, experiencing an intersection of social stigma and structural barriers that may threaten mental health and contribute to the acquisition of SUD (2, 3). It is, therefore no surprise that SUD affects nearly half of PLWH (4–8), with accompanying high rates of polysubstance use disorders, significantly higher than the general population (4, 5, 9–13). While there is no significant difference in SUD prevalence in White or Black populations, Black populations are less likely to receive treatment than White populations (19% vs. 24%) (14). Inadequate healthcare access, stigma, and criminalization related to structural racism exacerbate disparities in HIV and SUD outcomes (15, 16).

Despite its high prevalence and adverse effect on health outcomes, SUDs are both underdiagnosed and undertreated among PLWH, especially Black PLWH. Globally, only half of HIV care and treatment sites routinely screen for SUDs and refer to substance use treatment. Rates of SUD are increasing (17), and up to three-quarters of PLWH meeting SUD criteria may not receive treatment (18). In areas with high proportion of Black residents, there were lower rates of treatment centers in the community, creating barriers to SUD treatment initiation and completion rates (18–20).

Furthermore, the current standard procedure for SUD screening relies on PLWH attending scheduled HIV clinic visits, where they are screened in the waiting area or an exam room (21, 22). Inherently, this approach poses two key structural barriers to screening. First, PLWH who have comorbid SUDs are less likely to attend clinic appointments (23–27). Second, SUD screening is usually performed by a provider during clinic visits, which may have lower validity when compared to self-reported questionnaires, particularly among racial, ethnic, and/or sexual and gender minorities (28, 29). Stigma related to substance use among PLWH may decrease disclosure of substance use during clinic visits (30, 31). Therefore, the current strategy for diagnosing SUD in PWLH is limited because it only reaches patients who both attend clinic visits and willingly disclose symptoms, disproportionately missing PLWH with the epidemiologically highest likelihood of requiring treatment for SUD at baseline.

A potential alternative that may alleviate these barriers is screening for SUD outside of clinic visits, which can be accomplished using electronic patient portals. Patient portals are secure websites, or web-based applications, that give patients access to their health information from anywhere with a web connection (32). Portals can be enabled to send questionnaires to patients to complete before clinic appointments, or even when no appointments are scheduled as part of a population health approach to screening. Moreover, portals have become increasingly available globally. Access to patient portals varies by country, with healthcare systems in developed countries often providing greater levels of access (33–36). 90% of health care systems in the United States offer online portal access to patients (37, 38). PLWH and those with SUD have high levels of access to the internet and high interest in using portals (39–41). There is also evidence that PLWH may be more likely to disclose substance use when screened using technology vs. through interviews (42). In our preliminary work, we established a system for population health to perform portal-based assessments for depression. In the primary care setting, we demonstrated higher rates of depression screening and identification of patients with moderate–severe depression when screening was performed using patient portals (43). HIV clinicians and patients have shown interest in using patient portals (39, 40, 44, 45). Additionally, patients who used patient portals have fewer no-show appointments, greater satisfaction in their care, and better engagement in care (46–52). One cross-sectional study found that patient portal utilization was associated with 80% greater odds of antiretroviral treatment adherence (53).

### Overview of ePORTAL HIV-S

In this manuscript, we describe the protocol for a randomized controlled trial (RCT) component of a NIDA-funded project—the Achieving Equity in Patient Outcome Reporting for Timely Assessments of Life with HIV and Substance Use (ePORTAL HIV-S) study (1R01DA058965). The study seeks to achieve health equity in SUD screening and treatment among Black adults living with HIV by implementing interventions to decrease barriers to screening and treatment within a large, real-world medical home. First, we will develop a culturally tailored approach to increase patient portal use among PLWH in our health system via a community health worker (CHW)-led initiative. The CHW will train participants how to use the portal to access appointments, health histories, and test results, how to send messages, and how to respond to questionnaires. The RCT will determine the effectiveness of population health (portal+clinic visit) vs. usual (clinic visit) SUD screening among PLWH in an HIV clinic setting. Additionally, we implement a collaborative care model to connect patients who screen positive for SUD to care and effectively treat for SUD. Finally, we will disseminate ePORTAL HIV-S to other sites that provide care for PLWH. In this manuscript we focus on describing the intervention protocol for the randomized control trial (RCT) to determine the effectiveness of population health vs. usual SUD screening among PLWH in an HIV clinic. The RCT protocol paper adheres to SPIRIT (Standard Protocol Items: Recommendations for Interventional Trials) (See Supplemental material) (54). Results and protocols for the CHW-led initiative and collaborative care model are beyond the scope of the current manuscript and will be reported separately.

## Methods and analysis

### Overview

#### Study design

**Figure 1:**
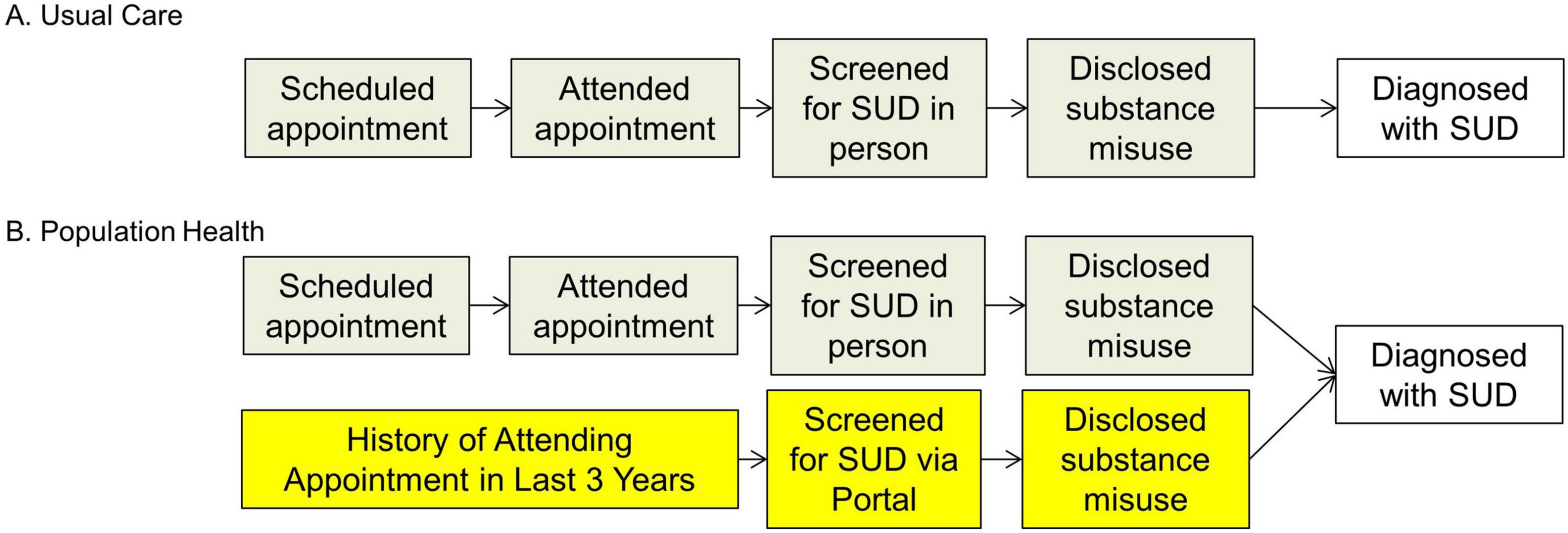
Clinic based screening vs. Population health screening. **(A)** Clinal based screening (current practice) and **(B)** Population health screening (new practice). SUD, substance use disorder.

We propose an RCT, to determine whether population health, patient portal-based SUD screening will increase screening rates and identification of SUD in PLWH. We will randomize our cohort into two arms: the control arm, who will receive usual care (SUD screening during clinic visits), and the intervention arm, who will be invited to complete SUD screening via the patient portal as well as during clinic visits as per usual care. Figure 1 shows the SUD screening process for the intervention arm vs. control arm. The RCT will be a one-year study, which allows sufficient time for the usual care arm to have an HIV care clinic visit and be screened for a substance use disorder. Patients will be randomized 1:1 in groups of 100.

### Selection/treatment of subjects

This study will be conducted at the University of Chicago Medicine (UCM) Ryan White Adult HIV Care Program. The UCM Ryan White clinic is the lead site for the Chicago Department of Public Health-funded South Side Health Home (S2H2), a major provider of HIV prevention and care services for residents of Chicago’s South Side, one of the epicenters of the United States HIV epidemic (55). It is currently staffed by 15 physicians, 8 fellows, 1 nurse practitioner, 1 licensed practical nurse, 2 pharmacists, and 2 licensed social workers. Eligibility criteria include adult (>18) patients living with HIV who receive care at the UCM Ryan White Adult HIV Care Program, and appear on the HIV care patient registry, who have not completed SUD screening in the prior 12 months, and who have an active portal account not managed by a proxy.

#### Sample size

**Figure 2:**
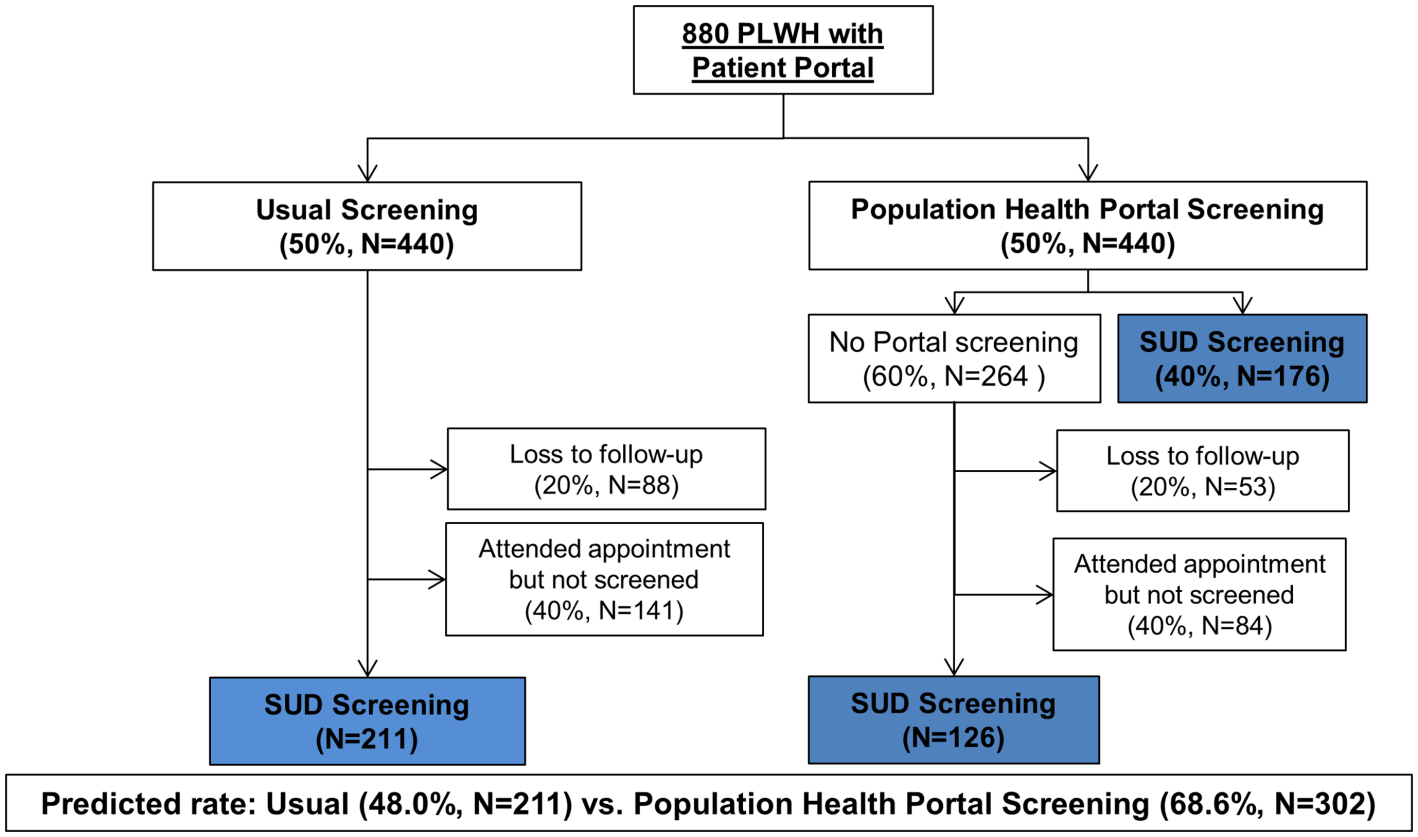
Flow diagram of planned participant enrollment and follow-up. PLWH, people living with human immunodeficiency virus; SUD, substance use disorder.

There are ~880 patients who will meet eligibility criteria; therefore, ~440 PLWH will be randomized to each arm. There is an approximately 80% retention in care rate in our clinic. For the usual care arm, based on the rates of depression screening at our clinic, we estimate the rate of in-clinic screening will be about 60%. For the intervention arm, we estimate that 40% of people will complete the portal screener, based on our data from the PORTAL-Depression study (56). Therefore, we anticipate the screening rates will be 48% in usual care and 69% in the intervention arm (Figure 2). Using a two-sided chi-square test to detect a difference in proportion between two independent samples, with an alpha of 0.05 we will have 99% power to detect this difference.

### Intervention

#### Development of ePORTAL HIV-S

ePORTAL HIV-S was developed by evaluating and adapting previously implemented successful methods of electronic mental and behavioral health screening and applying these to our specific patient population of PLWH. We first built on the PORTAL-Depression study (56), which developed a system for population health mental health assessments via the patient portal. We chose to use the NIDA Quick Screen V1.0 to screen for SUD in order to provide HIV providers with a thorough, evidence-based screener for patients (45). These studies led to adaptations of the PORTAL-Depression study for this intervention.

#### Screening protocol

Patients randomized to population health screening–the intervention arm–will receive an email invitation and/or mobile app message (depending on their preferences) to log into their portal account and complete the screener. Patients in this arm will receive invitations regardless of whether they have a scheduled appointment. The language in the email invitation/mobile app message is standard to all portal-based messages from UCM, encouraging patients to log in to their portal account to read a message. Once the message is opened, patients will be invited to complete the SUD screener. The SUD screener opens with a brief paragraph that serves to normalize and de-stigmatize screening by explaining that these questions are a part of standard clinical practice in the HIV clinic, and that a research study is being conducted regarding screening via the electronic patient portal. Electronic informed consent is obtained. If patients consent, they can complete the screener through the patient portal. Once completed, screening results will be automatically uploaded into a flowsheet in the electronic health record (EHR). Patients may choose to ignore screening invitations, leave the study by notifying their provider, or only partially complete the tool. Additionally, some of these patients may have clinic visits during the study period and could receive screening as part of their usual clinical care. Patients in the usual care arm will be offered SUD screening during clinic visits using the NIDA Quick Screen V1.0. Medical Assistants (MAs) will offer SUD screening to PLWH as a routine practice after directing them to a clinic room. Medical providers may also administer the screening if MAs do not do so. A best practice advisory in the electronic health record alerts MAs and other providers if a patient is due for screening, i.e., has not completed SUD screening in the prior 12 months.

For both the intervention and control arms, if a person has a NIDA Quick Screen score >3, indicating moderate to high risk of SUD, the EHR will automatically send a message to our collaborating CHW, infectious disease social worker, and the patient’s HIV clinician informing them of this result. The CHW will then contact patients who screen positive either in person during clinic visits or by telephone to follow-up and link patients to treatment. If they cannot reach patients by phone, they will send portal messages to engage patients in care.

#### ePORTAL HIV-S training/education

Prior to implementation of ePORTAL HIV-S, all medical staff, including clinicians, social workers, CHW and MAs will be informed of the initiative and the contents and administration of NIDA Quick Screen V1.0 via in-person training sessions (including faculty meetings and clinical operations meetings). The ePORTAL HIV-S team will monitor screening and linkage to care rates to provide dynamic assistance throughout the project and to ensure fidelity to study protocols.

#### Randomization

We will randomize patients at a 1:1 ratio to either population health or usual SUD screening (Figure 2). Randomization will be performed via computer-generated simple randomization. Concealment will be maintained via an electronic system. A staff member in the Center for Research Informatics at the University of Chicago who is not a member of the research team will be responsible for randomization and enrollment. Patients randomized to the population health screening arm will not be blinded to the study, since they will receive an email notification to complete a questionnaire in their portal account. Patients randomized to usual screening will not receive email notification, and thus, will be blinded to trial assignment. Clinicians will not be informed of the randomization; however, some patients may inform them of their assignment. If patients screen positive for a SUD, clinicians will receive notification of these positive results, which could lead to unblinding. However, the study data analysts who will be assessing outcomes will remain blinded.

#### Implementation plan

We will inform all HIV clinicians (attending physicians, fellows, and advanced practice nurses) in the UCM Ryan White clinic about the study via email and at regular clinic meetings. Clinicians can opt-in to the intervention via email. Clinicians who do not provide assent will have their patients excluded from the RCT. Once launched, the ePORTAL HIV-S staff will meet regularly to monitor screening rates, discuss patient engagement, data collection, and identify issues related to workflow. We will provide feedback to clinical leadership and MAs regarding rates of screening and linkage to care (Table 1).

**Table 1:** Schedule of patient enrollment, interventions, and assessments for the trial period.

	Trial period			
	Enrollment		Post-randomization	Close out
Time point	-2 weeks to 0	0	1 to 12 months	12 months
Enrollment				
Eligibility screen	×			
Randomization	×			
Informed consent		×		
Intervention or comparator				
Population health SUD screening		×	×	×
Usual care SUD screening		×	×	×
Assessments				
Age, sex, race, ethnicity	×	×		
Number and percentage screened for SUD				×
Number and percentage diagnosed with SUD				×
Number and percentage reffered for SUD treatment				×
HIV viral suppression				×
Retention in care, kept visit propotion				×

#### Intervention outcomes

The primary outcome is the percentage of PLWH who have had a clinic visit in the last two years in the Ryan White Clinic and who were screened for SUDs. Secondary outcomes include the number and percentage of PLWH diagnosed with SUDs, and the number and percentage of PLWH referred for SUD treatment. Secondary HIV care outcomes will include (1) retention in care measured as the kept visit proportion (the number of clinic visits attended divided by the number of clinic visits scheduled) in the 12 months after screening, and (2) HIV viral suppression, defined as a quantitative viral load <200 copies/mL in the year post-screening.

#### Data collection

Data will be stored in the electronic health record. To protect participant confidentiality, only limited data (deidentified except for dates) will be used for analysis. Data will be exported and deidentified by the Center for Research Informatics before it is securely transferred to the research team for analysis.

#### Ethics statement and dissemination

This study was approved by the University of Chicago Biological Sciences Division Institutional Review Board (IRB24-0684). The study was registered on 2024-09-27 with clinicaltrials.gov (NCT06682468). Patients will be consented for participation. Data will not be shared because it includes dates and is not completely deidentified, in accordance with institutional policy. Results from the study will be disseminated through conference presentations, peer-reviewed publications, and community reporting.

#### Data safety and monitoring

A Data Safety and Monitoring Board (DSMB) will be formed to protect the safety of participants. The DSMB will evaluate the implementation of the trial, retention rates, patient safety, and maintenance of the integrity of data collection and management during this study. Any and all serious adverse events will be forwarded to the DSMB within 48 h of being recognized by study staff. The DSMB will have authority to recommend modifications to the clinical investigations or to stop these investigations if there are concerns with patient safety or the integrity of the study. The DSMB will meet every six months for the duration of the clinical trial.

### Data analysis

The intention to treat principle will be applied to all analyses (57). For all data we will use descriptive statistics to characterize and describe both the control and intervention arms. To confirm successful randomization, study arms will be compared with regards to age, sex and race/ethnicity distribution. Additionally, we will confirm that differences between the two arms do not exist for type of insurance and major comorbidities. If data is missing for variables under comparison to determine successful randomization, we will report the number of missing responses for each and show differences in between individuals with complete data and those with incomplete data across intervention arms, multiple imputation using the chained equations (MICE) method. Finally, logistic regression models will be used to adjust for baseline imbalances between groups. Comparisons will be made for categorical variables using a chi-square test or Fisher’s exact test, and for continuous variables, a two-sample t-test or Wilcoxon rank-sum test. We will use Bonferroni correction during our examination of baseline differences between treatment and control groups if analyses suggest differences between the groups, we will present adjusted model results rather than unadjusted results.

For primary and secondary outcomes that are proportions or binomial outcomes (percentage screened for SUD, number diagnosed with SUD, percentage diagnosed with SUD, number referred for SUD treatment, percentage referred for SUD treatment, HIV viral suppression, retention in care and kept visit proportion) a chi-square test or Fisher’s exact test and beta regression models will be used to compare the proportional outcomes in the trial arms. All significance testing will be two sided and use a level of 0.05.

## Discussion

PLWH are disproportionately affected by comorbid SUDs, which leads to negative health outcomes, potentiated by the fact that SUDs are underdiagnosed and often not treated among PLWH. Currently, limited evidence exists describing the optimal screening strategy for SUD among PLWH. Our approach will be one of the first to incorporate the privacy and convenience of a patient portal-based SUD screener compared to the current standard of care: in-person screening during routine clinic visits. Our method allows patients to self-report data, which then is automatically relayed to the patient’s primary HIV clinician, thus potentially increasing sensitivity of screening and both standardizing and improving documentation of screening results. Furthermore, we are screening for SUD among a population of PLWH who have historically received care at the clinic, as a population health approach, which differs from the reactive approach of screening during visits. This will allow the opportunity to reach PLWH who may not attend clinic visits. Also, because ePORTAL HIV-S is being implemented pragmatically within a large, real-world medical home, it allows us to test our intervention’s effectiveness and implementation strategy within the context of existing clinical practice. SUD remains one of the biggest challenges facing healthcare today, across diverse populations (15, 58, 59). We believe that the results of this study–and the fundamentals of ePORTAL HIV-S, including confidential, convenient online screening and incorporation of a CHW–will be potentially applicable to broader populations at risk for SUD. It will provide crucial information regarding the effectiveness of portal-based screening for SUD. Our findings may also have implications for screening for other behavioral or mental health conditions, or other conditions that may be associated with stigma.

## Limitations

Our findings may not be generalizable in settings where there are significant differences in cultural mores related to substance use, HIV, and stigma compared to ours. Additionally, our findings are contingent on patients’ access to private personal electronic devices with internet access. Finally, our health system has access to behavioral health services, a CHW, and clinician collaboration, which may not generalize to other settings, particularly ones without access to EHR technology or low-resource medical settings, including other countries.
